# System of Human Physiology

**Published:** 1839-10

**Authors:** 


### Art. XII.

1. *Lehrbuch der Physiologie des Menschen.* Von Dr. Friedrich Arnold, Professor an der Hochschule in Zürich. Erster Thiel.—*Zurich,* 1836, pp. 388. Zweiten Theiles, erste Abtheilung.—*Zurich,* 1837. pp. 460.

*System of Human Physiology.* By Dr. Frederic Arnold, Professor in the University of Zurich. Vol. I.—*Zurich,* 1836. Vol. II. Part I.—*Zurich,* 1837.

2. *Lehrbuch der pathologischen Physiologie.* Von Dr. Wilhelm Arnold, Professor an der Hochschule in Zürich. Erster Thiel.—*Zurich,* 1836. Zweiten Theiles, erste Abtheilung.—*Zurich,* 1837, pp. 542.

*System of Pathological Physiology.* By Dr. W. Arnold, Professor in the University of Zurich. Vol. I.—*Zurich,* 1836. Vol. II. Part I. —*Zurich,* 1837.

In an article on the German school of physiology in our Ninth Number, we had already occasion to allude to the works of which the titles are prefixed, but the extensive nature of the subject prevented us at that time from examining them so minutely as their merits perhaps required. We have been since waiting for the completion of the works to renew our notice of them; but as so much time has already passed, we now propose to give a brief account of their contents, in their present unfinished state.

The reputation of Frederic Arnold, although of no ancient date, may already be termed European; and the name that he has acquired by his publications on the Eye and Nervous System will be amply sustained by the work now before us. The first volume is dedicated solely to the consideration of the general doctrines of the science. If this is a department which is too much neglected in British elementary works, Professor Arnold has erred by passing into the opposite extreme. There would seem to be this difference in general between the German and British mind, that whilst the former deems it necessary first to lay the broad foundation of general laws before proceeding to raise the detached structures, the latter at once commences with facts, and leaves the student to draw his general conclusions from the details which have been placed before him. The reader, therefore, who is at all conversant with general science, will be apt, on perusing Dr. Arnold's first volume, to think that much superfluous matter, relating to general physics and natural history, has been introduced, most of which, he will be inclined to say, cannot fail to be known to every well-educated general student.

In the first section of his first volume Dr. Arnold considers, 1st, the general physical and intellectual organization of man; 2dly, the differences between man and the brutes; and 3dly, the distinctive features by which the various races of mankind are physically and mentally characterized. In discussing the last topic, he adopts an arrangement of his own, somewhat resembling that of Bory de St. Vincent, and divides mankind into five great races, comprehending numerous subdivisions: I. The Caucasian race, embracing the Europeans and their descendants, the Western Asiatics and North Africans. II. The Altaic race, so called from the Altai mountains, embracing the northern, eastern, and southern Asiatics, with the exception of the Malays. III. The Indian-Oceanic race. IV. The Ethiopian race. V. The Aborigines of America. The minute anatomy of the tissues, which is fully and carefully given, concludes the first section.

The second section comprehends the relations of man to the external world, and treats firstly of the general physics of the earth and planetary system; noticing, for example, the effects produced upon man by various climates, by a pure or impure atmosphere, by proximity to the sea, by high or low-placed habitations, sudden changes of temperature, electricity, &c. It then treats of the organization on the surface of the earth, in so far as the wants of man are concerned, and under this head embraces a chapter on general dietetics. The third and concluding section refers to the general phenomena and laws of the living body, and comprehends definitions of contractility, irritability, elasticity, temperament, animal heat, animal electricity, sleep, &c.

The second volume bears the stamp of industry and intelligence impressed upon every page, and forms an excellent compendium of that part of modern physiological science of which it treats. It comprehends the processes of digestion, respiration, and circulation, and is written in a concise and distinct style. The opinions of others when differing from the author's are fairly stated, and the facts which appear to support the one or other side faithfully laid before the reader. In treating of digestion Dr. Arnold dwells largely on the interesting and important experiments of Beaumont and Eberle, but he does not seem to have been acquainted with the later experiments of Müller and Schwann, noticed in our Seventh Number. We shall extract some interesting observations from this part of the volume. After quoting the numerous and contradictory experiments as to the influence of the tenth pair of nerves (the eighth of other authors), on the process of digestion, he brings forward the following experiments, as calculated to throw some additional light on the subject.

"The experiments were performed on fowls and pigeons. In some instances the tenth pair was simply divided, but in others a piece of the nerve was cut out, and again in others the pharyngeal branches of the ninth and twelfth pair were cut in addition. The animals were kept fasting for two or three days before the operation, after which they were fed, and then allowed to live till they died in consequence of the injuries they had received. The crop, on examination, was found filled with grains of wheat; they were much swollen, and easily crushed by pressure, and were surrounded by a considerable quantity of chyme-like fluid, with a strong acid reaction. Wheaten bread which had been taken was found almost entirely chymified in the crop of a fowl. The proventriculus contained a slightly acid mucous fluid, and the gizzard a like fluid, with a stronger acid reaction, and also some grains of corn very much swollen, besides some husks and a considerable number of small pebbles. In the duodenum the fluid was more or less acid. Deglutition was not impeded by the operation, but the contractile powers of the proventriculus were considerably weakened, for in a fowl which lived for two days after the operation 329 grains out of 400 which had been swallowed, were found after death in the crop; and in a pigeon which lived for 52 hours, of 290 grains only 20 were missing. In two pigeons, in which, besides the pneumogastric nerves, the pharyngeal branches of the ninth and twelfth pairs had been divided, 29 grains were missing in the first, which lived 80 hours, out of 300 grains which had been swallowed, and in the second, which lived 66 hours, 45 grains were missing out of 350. In these cases no grains or husks could be detected in the gizzard, although in the other cases in which the pneumogastric nerves only had been cut, both grains and husks were found in that organ.

"The secretions of the crop are not suppressed by the division of the above-mentioned nerves; for the grains, although they had diminished in number, had acquired considerable addition in weight; in the crop of a pigeon, with a loss of 20 grains in number, there was a gain of 50 grains in weight, the animal having lived 52 hours; in a fowl with a loss of 71 grains in number, there was a gain in 48 hours of 164 grains in weight; and in another where there was a loss of 45 grains of wheat, there was a gain of 261 grains in weight in 66 hours; and in a third a gain of 335 grains in weight in 80 hours, with a loss of 29 grains in number. The animals did not appear to die from starvation, but from the effects of impeded respiration. It results from these experiments, that the secretion of an acid fluid in the crop, proventriculus, and gizzard, is not suppressed by the division of the pneumogastric nerves, nor, judging from the quantity of fluid, would it even appear that it is lessened in quantity. The chymifying properties of the fluid remain the same as where there is full integrity of the pneumogastric nerves; and digestion, in so far as it is dependent upon the gastric juice, is not destroyed by the operation. The contractile powers of the proventriculus, however, are weakened, although neither they nor the tributary powers of the gizzard are completely destroyed. It appears, likewise, that simple division of the nerve is followed by the same consequences as excision of a part of its substance. The nerves of the stomach, therefore, appear to exert an influence upon chymification, in so far as this process depends upon the various motions of the organ." (p. 80.)

The following observations on the gradual conversion of the chyle into blood are equally interesting:

"The mucous membrane of the alimentary canal imbibes the fluid contained in the intestine precisely on the same principle as a sponge becomes saturated with water. The lacteal vessels do not, as has frequently been asserted, present open mouths upon the surface of the membrane, but they ramify through its substance along with the capillary blood-vessels. The structure of the walls of these capillary blood-vessels would appear to differ from that of the walls of the lacteals, and this difference affords the reason why the chyle passes into the lacteals only, and is not taken up, like the other fluid contents of the intestine, by the capillary vessels. The transformation of the chyle into blood probably commences in the tissue of the mucous membrane; a reciprocal action takes place in the substance of the membrane between the blood and chyle, and certain principles of the former are imparted to the latter, such as the red colouring matter, fibrine, and perhaps alkali. This constitutes the first change in the chyle, and disposes it to form globules. The chyle assumes more and more the characters of blood with each mesenteric gland through which it passes. The same reciprocal action takes place here, the red colouring matter and fibrine pass from the blood into the chyle; and it is probable, too, that the blood parts with some of its oxygen, by which the albumen of the chyle becomes changed into fibrine." (p. 149.)

"It is probable, from what has already been said, that the formation of the globules of the chyle commences in the lacteal vessels during the process of absorption. At least the examination of chyle taken from the lacteal vessels close to the intestinal canal corresponds, in the small quantity of globules which it contains, with the observation that it does not coagulate before its passage through the mesenteric glands, and that its coagulating power and the number of its globules become greater the nearer it reaches the extremity of the thoracic duct. Besides, there is no sufficient evidence to show that fibrine is formed in the intestinal canal and passes from it into the chyle; this opinion, on the contrary, is opposed by the facts already noticed, and it is rendered improbable by the consideration that the fibrine of the aliment ceases to exist, as such, in the chyme, but is changed into albumen or some other animal principle. The number of the globules of the chyle increases with its progress in the lacteal vessels, a fact which the examination of a drop of the fluid before and after its passage through the mesenteric glands, or in the lower or upper portion of the thoracic duct, readily proves. The chyle, when it has reached the extremity of the thoracic duct, is very rich in globules. These are not all alike, some of them being one third in diameter less than others. The smaller are perfectly globular, but the others, from their central depression and marginal ring, as likewise from their chemical relations with water, appear to be identical with the globules of the blood. Some of the latter, however, when examined singly beneath the field of the microscope, show no appearance of the red colouring matter, but this appearance of individual globules is not necessary to prove that the red colour of the chyle depends upon the globules of the blood. A careful examination of the chyle proves further that the red colouring matter does not occur in it in a dissolved state, but, as in the blood, united with the globules; a fact which I have had occasion to observe, not only in the coagulum, but also in the serum of the chyle of dogs, the red colour being quite sufficient to point out the presence of cruor even to the naked eye. The supposition, therefore, that the colouring matter occurs in the chyle in a dissolved state, is unfounded. The circumstance that the chyle, and also the lymph which has been in relation with the blood through the medium of the lymphatic glands, contain, besides the completely round and smaller lacteal globules, other larger globules, which correspond in size and shape with those of the blood; the observation that the number of the latter augments with the progression of the chyle in the lacteal vessels, whilst the number of the former undergoes a relative diminution; and lastly, the fact that the central nuclei of the larger globules correspond in size and shape with the smaller, leave no doubt that the globules of the blood are formed from the smaller globules of the lymph and chyle, which, from their affinity to the red colouring matter, attract this substance from the blood with which they come in indirect contact, through the walls of the capillary vessels." (p. 174.)

If these views be correct, the globules of the chyle must always stand in a definite relation to the globules of the blood. But this assumption is combated by many able physiologists. According to Müller, the lacteal globules are sometimes equal in size to those of the blood, as in the cat; in other instances, as in the dog and goat, they are rather smaller; and in others again, as in the rabbit, much smaller than those of the blood. Hewson found the lacteal globules in man resembling in size and shape the central nuclei of the globules of the blood, and Prevost and Dumas give their size as rather more than half the size of the globules of the blood.

In the chapter on Respiration, Professor Arnold strongly expresses his dissent from Sir Charles Bell's views as to the respiratory tract of the spinal cord.

"It has been supposed," he says, "that the nerves subservient to respiration form a peculiar system, that of the irregular nerves, in opposition to the system of regular nerves on which motion and sensation are dependent. To this system have been reckoned to belong the third, fourth, and sixth pairs for the eye; the twelfth for the tongue; the ninth for the pharynx; the seventh for the face; the tenth for the larynx, heart, lungs, and stomach; the phrenic for the diaphragm; the accessory of Willis for the humeral muscles, and the external respiratory for the thorax. They are distinguished by their origin by one root only, and by having no ganglion. According to this hypothesis, the seventh, ninth, tenth, and eleventh pairs of cerebral nerves, the phrenic and external respiratory nerve bring the lungs and respiratory muscles into mutual relation, and arise in one line, forming a particular column of the spinal marrow, which lies between the olivary bodies and crura cerebelli, and which may be traced along the lateral aspect of the spinal marrow between the grooves, for the anterior and posterior roots. These nerves may be cut, and thus several muscles be prevented from taking part in the act of respiration, without impairment of their voluntary motion. Thus when the eleventh and seventh pairs are cut, the respiratory motions only of the humeral and cervical muscles are interrupted, but the power of voluntary motion which they derive from other means is not destroyed. To this hypothesis, which easily deceives by its simplicity, but which on nearer examination presents many errors and discrepancies, it may be objected that the respiratory and voluntary motions are allied, and by no means opposed to each other; and that many of the so called regular nerves take part in the function of respiration, as for example, all the thoracic nerves; secondly, that the origin of the irregular nerves from a lateral tract is not only not proved, but that it is, in the case of several of them, unsusceptible of proof; thirdly, that some of these nerves, as the ninth and tenth pair, have ganglions; and lastly, that it does not depend upon any specific property of the nerve whether such or such a motion is produced, but whether the nerves supply this or that muscle." (p. 220.)

Innumerable theories have been advanced to explain satisfactorily the nature of the changes which the blood and air undergo during the act of respiration. Another has been propounded by Professor Arnold, which, in many of its leading features, corresponds with that advocated by Tiedemann, Gmelin, and Mitscherlich.

"As it is more than probable that the carbonic acid occurs in the venous blood, united with some substance from which it is separated with greater or less rapidity by the contact of the atmospheric air, and as, further, the carbonate of the protoxide of iron greedily withdraws oxygen from the atmosphere, at the same time parting with its carbonic acid and becoming changed into a peroxide, it may reasonably be supposed that the carbonic acid of the venous blood is united with the iron of the red colouring matter, and that it is set free during the act of respiration, by the reciprocal action of the blood and air. The protoxide at the same time, by absorption of oxygen becomes a peroxide, which, during the circulation of the blood through the capillaries, again parts with its oxygen. Carbon is at the same time eliminated from the blood, and unites with the liberated oxygen to form carbonic acid, which is thrown out by the lungs, whilst oxygen is again absorbed." (p. 252.)

This theory has likewise been advocated by Maack, in his treatise "De ratione, quæ colorem sanguinis inter et respirationis functionem intercedit," where various chemical experiments are adduced in support of it.

We have given the above extracts chiefly as specimens of the work. Throughout the whole volume we find a careful selection of facts, with cautions and in general just reasoning. It contains, besides, the results of various of Dr. Arnold's own experiments, which are generally important. In criticising the opinions of others, Professor A. shows, with one exception, the strictest impartiality; but when speaking of Müller, of Berlin, he occasionally displays a spirit of ill-disguised jealousy.

We may here remark, that the works of the two brothers are written upon the same plan, and embrace the same number of sections and chapters; but Frederic treats only of healthy, and William of pathological physiology. The volume on general doctrines, prefixed to the pathological physiology, by the latter, is burdened with all the faults already remarked in the corresponding volume of his brother's work, whilst it possesses few of its redeeming qualities. The statements are so lax and general, that the reader rises from their perusal with no definite or fixed impression. In the first section, on the organization of man in the diseased state, with reference to general deviations from the healthy standard, we find some remarks on the moral and intellectual faculties; on the modifications they undergo by sex, age, climate, and disease; on the distinctive characters of man and the brutes; and on the characteristics of giants and dwarfs. The remarks on the diseases of the different tissues may be useful, by rendering the beginner familiar with their names, but otherwise they can be of little value. The second section comprehends observations on the influence of the sun, moon, and planets; of light, heat, cold, magnetism, and electricity; and of the various winds in producing and modifying disease. Some of the remarks in this chapter are sufficiently curious, but, as usual, deficient in precision. The following passage will startle some of our cautious readers.

"The relation of the moon to the earth, and its influence on man, and on the production of human disease, have in all ages furnished copious matter for controversy. Whilst such influence has been totally denied by some, by far too much has been referred to it by others, whose assertions, however, are not always borne out by facts. But it results from incontrovertible facts, that the cause and aggravation of many diseases depend upon the lunar influence. At change of moon, chronic swellings, particularly those of glands, as of the thyroid, vermicular affections, and many other diseases, decrease or become aggravated. On it depends the origin and crisis of many diseases, particularly of fevers; and these relations of cause and effect are said to be very manifest within the tropics. At new moon nervous diseases, such as epilepsy, chorea, somnambulism, often become aggravated, and their attacks are then more frequent and severe. In the wane, many tumours and vermicular affections are said to yield to remedies much more readily than at other times, an opinion which is in general repute with the vulgar." (p. 96.)

He thus speaks of the effects of magnetism:

"On the application of the north pole of the magnet, the patient becomes aware of a prickly, vibratory, pulsating sensation in the point of contact, of a determination of blood to the part, and of a feeling of heat or cold. If of a melancholy disposition, he often complains of great uneasiness, sleepiness, or general soreness. Febrile symptoms occasionally ensue, with heat, sometimes of the whole body, sometimes only of a single part, as of the head, whilst the extremities may be cold. Pain in different parts of the body also frequently follow, such as toothach or headach, accompanied in some cases by giddiness, impaired memory, or even loss of consciousness; by irritation, or even deception of the organs of the senses." (p. 106.)

It would not be difficult, on reading these quotations, to suppose that they were extracts from some author of the middle ages, instead of being the production of an existing professor of the university of Zurich. In another part, we are cautioned against the immoderate use of snails and oysters, as, by furnishing too large a supply of material for the manufacture of semen, they may exert a deleterious influence on the genital organs. The whole volume abounds with crude notions of a similar description, which detract greatly from its value, as it is impossible for the beginner to separate the chaff from the wheat.

The third section, which treats of the more prominent general symptoms of disease, such as increased or diminished irritability, increased or diminished animal caloric, of the days of crisis, &c. contains nothing which calls for notice.

We have very little to say of Professor William's second volume. He goes over the same ground with his brother, and notices the pathological changes in the structure of organs, the consequent disorder of their functions, and other abnormal phenomena. The work is more characterized by plodding industry than original talent; it is almost entirely a compilation, and is open to one grievous objection, that as much time, space, and care are devoted to the most trivial subjects as to the most important doctrines of pathological science.

---

